# Curcumin and Biochemical Parameters in Metabolic-Associated Fatty Liver Disease (MAFLD)—A Review

**DOI:** 10.3390/nu13082654

**Published:** 2021-07-30

**Authors:** Gracjan Różański, Sławomir Kujawski, Julia L. Newton, Paweł Zalewski, Joanna Słomko

**Affiliations:** 1 Scientific Research Club of Exercise Physiology at Department of Exercise Physiology and Functional Anatomy, Ludwik Rydygier Collegium Medicum in Bydgoszcz Nicolaus Copernicus University in Torun, M. Sklodowskiej-Curie 9, 85-094 Bydgoszcz, Poland; 2 Department of Exercise Physiology and Functional Anatomy, Ludwik Rydygier Collegium Medicum in Bydgoszcz Nicolaus Copernicus University in Torun, M. Sklodowskiej-Curie 9, 85-094 Bydgoszcz, Poland; skujawski@cm.umk.pl (S.K.); p.zalewski@cm.umk.pl (P.Z.); jslomko@cm.umk.pl (J.S.); 3 Population Health Sciences Institute, The Medical School, Newcastle University, Newcastle-upon-Tyne NE2 4AX, UK; julia.newton@ahsn-nenc.org.uk

**Keywords:** MAFLD, curcumin, supplementation, liver

## Abstract

Metabolic-associated fatty liver disease (MAFLD), formerly non-alcoholic fatty liver disease (NAFLD), is characterized by excessive fat accumulation in hepatocytes. It is the most common chronic liver disease worldwide and is a significant public health problem. In the absence of pharmacological therapy, other treatments such as diet, physical activity, or supplementation are sought. Non-pharmacological therapies may include curcumin supplementation, which has been shown to have many health-promoting properties, including antioxidant, anti-inflammatory, and anti-cancer effects. For this reason, we reviewed available databases to analyze publications describing the effect of curcumin supplementation on biochemical parameters in MAFLD. Nine studies (eight RCTs and one CT) based solely on supplementation of patients with curcumin were included in this review. The results from the individual trials were varied and did not allow clear conclusions. Although they suggest that curcumin shows some potential in the treatment of MAFLD, further research is needed.

## 1. Introduction

Metabolic-associated fatty liver disease (MAFLD), formerly non-alcoholic fatty liver disease (NAFLD), is the most common chronic liver disease worldwide [1,2,3,4,5,6,7,8]. It is defined as a fat content of hepatocytes greater than 5% of the total weight of the liver, not caused by alcohol consumption, medication, or viral infection [9]. The prevalence of NAFLD has increased significantly in recent years, making it a serious public health problem. In 2013, Welsh et al. reported that up to half of obese men suffer from the disease [10]. Apart from obesity, MAFLD is often associated with other diseases such as insulin resistance, diabetes mellitus, metabolic syndrome and dyslipidemia [11,12,13,14,15]. In addition, it is also associated with an increased risk of liver- and cardiovascular disease-related mortality [16,17]. MAFLD is also associated with the risk of non-alcoholic steatohepatitis (NASH). It is estimated that 23–44% of MAFLD patients will develop NASH, which in approximately 37–41% of cases will lead to fibrosis, with 10–20% of these going on to develop cirrhosis. Within 5–7 years, cirrhosis will lead to liver failure in 40–60% of people, and hepatocellular carcinoma (HCC) in 2.4–12% of patients within 3–7 years [18]. The annual medical costs related to MAFLD have been estimated at €89 billion in the US and €35 billion in Europe. Early diagnosis, prevention, and treatment of risk factors as well as lifestyle modifications have been proposed as a cost-effective treatment strategy for MAFLD [19]. In 2016, the European Association for the Study of the Liver recommended the use of interventions leading to lifestyle changes in patients with MAFLD, specifically dietary changes and a gradual increase in aerobic exercise or resistance training [20].

### 1.1. Pathophysiology: Multiple Hits Hypothesis

The development of MAFLD can be influenced by dietary habits and genetic and environmental factors, making the pathogenesis of the disease complex, multifactorial, not yet fully understood, and therefore now referred to as a "multiple hits hypothesis". The key factor leading to MAFLD is insulin resistance (IR), resulting in an increase in de novo hepatic lipogenesis (DNL) as well as weaker inhibition of adipose tissue lipolysis, which leads to an increased flow of fatty acids to the liver and their accumulation in hepatocytes in the form of triglycerides. IR also affects the dysfunction of adipose tissue, resulting in altered production and secretion of adipokines and pro-inflammatory cytokines. Lipotoxicity is increased due to high levels of free fatty acids, free cholesterol, and other lipid metabolites, leading to the production of reactive oxygen species resulting in dysfunction of the mitochondria and endoplasmic reticulum. Moreover, changes in the intestinal microbiota leading to increased permeability of the small intestine, which results in greater absorption of fatty acids and increased levels of circulating molecules, have also been described as being involved in the pathogenesis. As a result, inflammatory pathways are activated and pro-inflammatory cytokines such as IL-6 and TNF-α are released [15].

### 1.2. Curcumin

Curcumin is a polyphenol classified as a curcuminoid. Its source of origin is turmeric (*Curcuma longa*), a plant from the ginger family found in Asia, mainly in India, where it is most often used as a spice due to its taste and aroma and intense yellow color. However, beyond its culinary use, it is valued for its health-promoting properties, with a history of use dating back several thousand years [21]. It has antioxidant, anti-inflammatory, and anticancer effects, among others, largely without adverse effects. Therefore, its use is being explored in the course of many diseases, such as allergic asthma; cardiovascular diseases; cancers of the lung, breast, colon, pancreas, and stomach; inflammatory bowel diseases; diabetes; and liver diseases [22,23,24,25,26].

## 2. Materials and Methods

### 2.1. Types of Participants

Studies were included if they were conducted in adult patients (aged > 18) of any gender or nationality with metabolic-associated fatty liver disease.

### 2.2. Types of Interventions

Interventions using curcumin supplementation alone, and presenting the results of biochemical parameter outcomes before and after supplementation, were included. Studies that recommended dietary changes and/or physical activity in addition to supplementation and animal trials were excluded.

### 2.3. Types of Comparisons

No specific comparison criteria were applied.

### 2.4. Types of Outcomes

Outcomes for at least one biochemical parameter were presented in the study, measured at baseline (pre-supplementation) and at post-supplementation.

### 2.5. Types of Studies

Any type of study (apart from case reports and reviews) was included if it was a study published in peer-reviewed journals in English. There were no restrictions on intervention length or follow-up measurement points. Exclusion criteria were as follows: non-human studies, use of an additional intervention beyond supplementation such as diet changes, lifestyle changes, or physical activity. The PICOS criteria for inclusion and exclusion of studies are shown in Table 1.

### 2.6. Search Strategy and Study Selection

**Figure 1:**
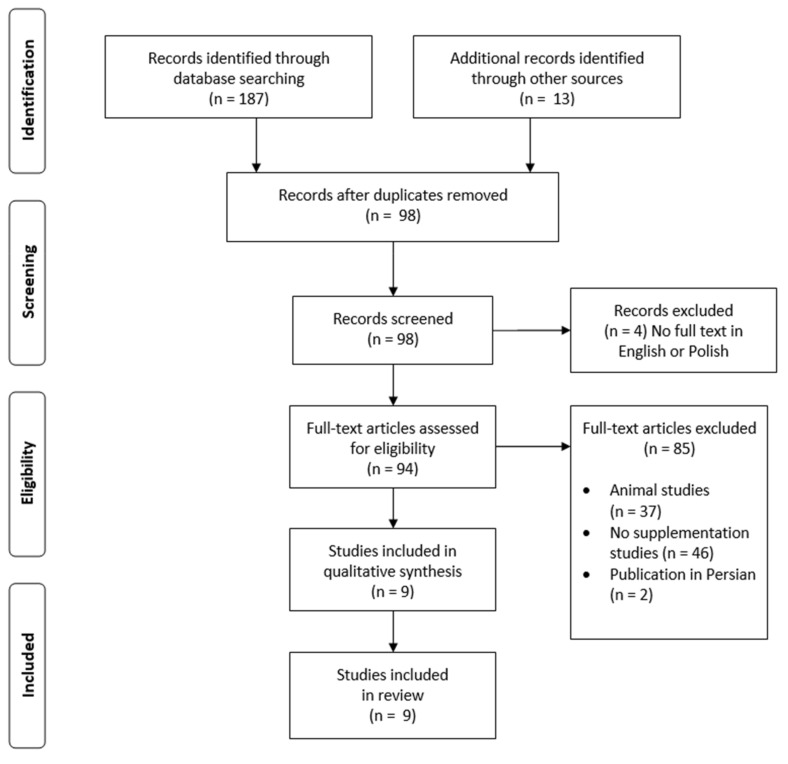
PRISMA flow diagram of the study selection.

We reviewed available publications using databases such as PubMed, Web of Science, and Scopus with the search words "NAFLD" or “MAFLD” or “metabolic-associated fatty liver disease” or "non-alcoholic fatty liver disease" and "curcumin" or "turmeric". We limited the results to papers in English or Polish (Figure 1).

### 2.7. Data Presentation

The network graphs were created using Cytoscape software version 3.8.1 [27].

## 3. Results

### 3.1. Study Selection

Nine studies (eight RCTs and one CT) that used only curcumin supplementation in MAFLD patients were included in this review.

### 3.2. Participant Characteristics

In most of the trials, the mean age of the patients was in the range of 37.41–49.4 years (Table 2) [28,29,30,31,32,33,34]. In one study, the average age of the respondents was 66.72 years [35], in another, the mean age of the study group was not given, but ranged between 18–70 years [36]. The average BMI values were in the 27.6–31.81 kg/m^2^ range.

### 3.3. Dosing and Duration

Differences in duration and doses of the supplement were observed between the interventions (Table 3). In three studies, the supplementation period lasted 12 weeks [29,30,35]; in the other studies, it was 8 weeks [28,31,32,33,34,36]. The daily supplement doses taken by patients in each trial were varied. Kelardah et al. [29,35] used 80 mg/day curcumin as nanomicelle in two interventions. Chashmniam et al. [31], Mirhafez et al. [32], and Hariri et al. [34] used phospholipid curcumin at 250 mg/day (equivalent to 50 mg pure curcumin). Saberi-Karimian et al. [36] used 500 mg curcuminoids plus5 mg piperine/day in their study, while [28] used 500 mg/day of an amorphous dispersion preparation comprising 70 mg curcuminoids. Panahi et al. [33] used 3 × 500 mg curcumin/day (100 mg curcuminoids per capsule). Ghaffari et al. [30] used the highest dose, which was 3 g/day turmeric (6 × 500 mg/day).

### 3.4. Supplementation and Biochemical Parameters

All nine studies monitored ALT and AST levels before and after supplementation [28,29,30,31,32,33,34,35,36]. Only Panahi et al. [33] (ALT: 40.7 ± 15.0 U/L vs. 22.0 ± 7.2 U/L, *p* < 0.001; AST: 35.4 ± 11.9 U/L vs. 22.6 ± 7.2 U/L, *p* < 0.001) and Rahmani et al. [28] (ALT: 39.07 ± 19.79 U/L vs. 36.08 ± 46.58 U/L, *p* < 0.001; AST: 28.88 ± 10.60 U/L vs. 23.84 ± 7.83 U/L, *p* < 0.001) observed a statistically significant improvement in these parameters

Chashmniam et al. [31] did not report statistical significance for any of the parameters examined, but also noted a decrease in ALT levels (50.08 ± 7.27 U/L vs. 43.28 ± 4.91 U/L) and AST (35.16 ± 3.9 U/L vs. 31.85 ± 3.41 U/L). All six of the other studies observed decreases in ALT levels after supplementation, but these were not statistically significant [29,30,32,34,35,36]. For AST, Ghaffari et al. [30] observed an increase in levels from 24 ± 11.5 U/L to 24.1 ± 8.90 U/L (*p* = 0.92); decreases were observed in the remaining studies, but only Panahi et al. [33] and Rahmani et al. [28] obtained statistically significant results (35.4 ± 11.9 U/L vs. 22.6 ± 7.2 U/L, *p* < 0.001 and 28.88 ± 10.60 U/L vs. 23.84 ± 7.83 U/L, *p* < 0.001, respectively).

Chashmniam et al. [31] and Kelardeh et al. (2017 and 2020) [29,35] also controlled for ALP levels, of which they noted decreases of 206 ± 14.45 IU/L vs. 197.6 ± 14.26 IU/L; 186 ± 13.6 IU/L vs. 182.6 ± 11 IU/L (*p* = 0.004), and 376.36 ± 88.45 vs. 376.09 ± 86.70 IU/L (*p* > 0.05), respectively.

Fasting glucose levels were controlled in four trials. Decreases were noted in all, but none of the decreases were reported as statistically significant [28,31,32,36]. Furthermore, Rahmani et al. [28] additionally measured HbA1c levels and obtained a statistically significant decrease (6.31 ± 1.62% vs. 5.53 ± 1.27%, *p* < 0.001).

TG, LDL-C, and HDL-C were tested in five trials [28,31,32,33,36]. Chashmniam et al. [31] noted an increase in the TG level (151.84 ± 15.46 mg/dL vs. 176.52 ± 14.5 mg/dL) and other authors indicated decreases, but only Panahi et al. [33] obtained a statistically significant result (164.2 ± 32.3 mg/dL vs. 129.2 ± 34.1 mg/dL, *p* < 0.001).

For LDL-C, Chasmniam et al. [31] and Saberi-Karimian et al. [36] reported increased levels after supplementation, respectively: 117.38 ± 6.04 mg/dL vs. 123.43 ± 7.09 mg/dL and 107.18 ± 39.48 mg/dL, change = 1.22 ± 19.75 mg/dL (non-statistically significant change compared to the change in the control group). Mirhafez et al. [32] indicated a non-statistically significant decrease (*p* = 0.15), while significant decreases were noted by Panahi et al. (149.0 ± 22.9 mg/dL vs. 101.3 ± 14.1 mg/dL, *p* < 0.001) [33] and Rahmani et al. (107.06 ± 31.36 mg/dL vs. 95.59 ± 28.22 mg/dL, *p* = 0.007) [28].

A decrease in HDL-C levels was reported by Chashmniam et al. [31] (45.84 ± 1.81 mg/dL vs. 43.03 ± 1.62 mg/dL) and Panahi et al. [33] (42.7 ± 4.4 mg/dL vs. 41.0 ± 4.0 mg/dL, *p* < 0.001). HDL-C levels increased in the other three studies, but only Rahmani et al. [28] obtained a statistically significant result (44.26 ± 11.83 mg/dL vs. 46.68 ± 28.22 mg/dL, *p* = 0.010).

TC levels were studied in four trials [28,31,32,36], while Panahi et al. [33] controlled for non-HDL-C levels. The authors of all four studies reported a decrease in TC levels after supplementation, but only Rahmani et al. [28] obtained a statistically significant result (198.59 ± 41.76 mg/dL vs. 174.38 ± 39.56 mg/dL, *p* < 0.001). Panahi et al. [33] reported a statistically significant decrease in non-HDL-C levels (157.5 ± 32.2 mg/dL vs. 104.1 ± 38.4 mg/dL, *p* < 0.001).

Panahi et al. [33] were the only ones to control uric acid levels in their sample and observed a statistically significant decrease (5.9 ± 0.6 mg/dL vs. 5.2 ± 0.5 mg/dL, *p* < 0.001).

Ghaffari et al. [30] and Saberi-Karimian et al. [36] also examined IL-6 and TNF-α levels. For IL-6, there were non-statistically significant decreases in levels in both studies. TNF-α also decreased in both studies, but only Saberi-Karimian et al. [36] obtained a statistically significant change (1.83 pg/mL, change = −0.20 pg/mL, *p* = 0.024) compared to the placebo group.

Chashmniam et al. [31] and Kelardeh et al. [35] controlled for total bilirubin levels. In both studies, decreases were observed, respectively: 0.94 ± 0.09 mg/dL vs. 0.72 ± 0.07 mg/dL and 1.25 ± 0.19 mg/dL vs. 1.21 ± 0.17 mg/dL (not significant). Chashmniam et al. [31] additionally measured direct bilirubin levels and noted a decrease (0.27 ± 0.02 mg/dL vs. 0.23 ± 0.03 mg/dL). Moreover, they also tested creatinine levels, of which they observed an increase (1.02 ± 0.02 mg/dL vs. 1.07 ± 0.03 mg/dL), and urea levels, which remained unchanged (32.3 ± 1.63 mg/L vs. 32.3 ± 2.21 mg/L).

Mirhafez et al. [32] were the only ones to control leptin and adiponectin levels and the leptin:adiponectin ratio. For all three values, they reported statistically significant differences after supplementation: leptin (23.21 ± 16.93 ng/mL vs. 17.85 ± 14.11 ng/mL, *p* < 0.001), adiponectin (14.35 ± 7.72 ng/mL–18.23 ± 9.75 ng/mL, *p* < 0.001) and leptin:adiponectin ratio (1.81 ± 1.90 vs. 1.12 ± 1.09, *p* < 0.001).

Ghaffari et al. [30] in their study tested MDA, hs-CRP, and TAC levels. They observed non-statistically significant decreases in MDA (303 ± 125 nmol/L vs. 291 ± 118 nmol/L, *p* = 0.09) and hs-CRP (1.94 mg/L (0.10, 10) vs. 1.59 mg/L (0.20, 4.30), *p* = 0.10), whereas there was a statistically significant increase in TAC (2.07 ± 0.39 mg/dL vs. 2.25 ± 0.41 mg/dL, *p* = 0.01).

**Figure 2:**
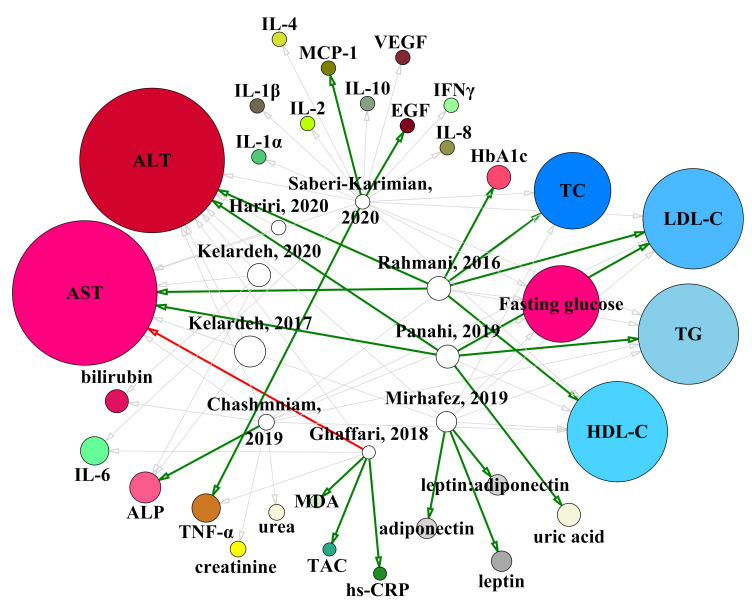
Network graph on effects of curcumin supplementation in MAFLD.

Saberi-Karimian et al. [36], in addition to IL-6 and TNF-α mentioned earlier, also controlled the levels of IL-6, TNF-α, IL-2, IL-4, IL-8, IL-10, IL-1α, IL-1β, IFNγ, MCP-1, EGF, and VEGF in their study. The only statistically significant changes in levels compared to the placebo group were for MCP-1 (288.23 pg/mL change = −75.22 pg/mL, *p* = 0.008) and EGF (107.2250 pg/mL, change = −10.42 pg/mL, *p* = 0.0001). Figure 2 summarizes results of the reviewed studies on the effects of curcumin supplementation in MAFLD.

Nodes denoting biochemical parameters on which effects of curcumin supplementation were examined are color-coded. Open circles denote reviewed studies. The size of the nodes (shown as dots) is proportionally related to the number of participants examined in a particular study/intervention group (for open nodes) or the overall number of participants on which the effect of curcumin supplementation on a particular parameter was examined (colored nodes). Edges (arrows connecting nodes) are shown in gray if the effect on a parameter noted in the study was not statistically significant. Thicker, green edges denote statistically significant improvement (health benefit), while red edges denote worsening of a parameter (deleterious health effect) noted in response to curcumin supplementation. ALP, alkaline phosphatase; ALT, alanine aminotransferase; AST, aspartate aminotransferase; EGF, epidermal growth factor; HbA1c, glycated hemoglobin; HDL-C, high-density lipoprotein cholesterol; hs-CRP, high-sensitivity C-reactive protein; IFNγ, interferon gamma; IL-1α, interleukin-1 alpha; IL-1β, interleukin-1 beta; IL-2, interleukin-2; IL-4, interleukin-4; IL-6, interleukin-6; IL-8, interleukin-8; IL-10, interleukin-10; LDL-C, low-density lipoprotein cholesterol; MCP-1, monocyte chemoattractant protein-1; MDA, malondialdehyde; non-HDL-C, non-high-density lipoprotein cholesterol; TAC, total antioxidant capacity; TC, total cholesterol; TG, triglycerides; TNF-α, tumor necrosis factor alpha; VEGF, vascular endothelial growth factor.

## 4. Discussion

To the best of our knowledge, this is the first review on the effects of curcumin on biochemical parameters in MAFLD focusing on supplementation-only interventions.

Curcumin is characterized by numerous pro-health (especially anti-inflammatory and antioxidant) properties. In addition, research has shown that it is able to influence many molecular mechanisms. Additional advantages are its natural origin and lack of serious side effects, indicating that it can be safely used in most patients. As a result, it is used in many diseases, such as cancer, neurological diseases, or inflammatory bowel diseases, as well as in the course of pathological changes in the liver [37,38].

In the disease course of MAFLD, there is increased production of reactive oxygen species and release of pro-inflammatory cytokines such as IL-6 and TNF-α [15]. Ghaffari et al. (3 g/day turmeric (6 × 500 mg/day)) [30] and Saberi-Karimian et al. (500 mg curcuminoids plus 5 mg piperine/day) [36] after 8 and 12 weeks of supplementation, respectively, reported a non-statistically significant decrease in IL-6. TNF-α levels also decreased in both studies, but only Saberi-Karimian et al. [36] obtained a statistically significant result. As mentioned earlier, curcumin also exhibits anti-inflammatory effects. It decreases the activity of the transcription factor NF-kβ, on which depend, among others: IL-1α, IL-1β, IL-2, IL-6, IL-8, IL-9, INFγ, and INFβ [37,39,40]. However, Gorabi et al. reported in their meta-analysis, which included 32 studies, that curcumin may show beneficial effects in reducing levels of IL-1 and TNF-α, but not IL-6 and IL-8 [41].

Ghaffari et al. [30], after 12 weeks of using the supplement at a dose of 3 g/day turmeric (6 × 500 mg/ day) recorded a statistically significant increase in TAC.

Curcumin also exhibits antioxidant properties comparable to vitamins C and E. It increases, among others, the activity of catalase, superoxide dismutase (SOD), glutathione peroxidase (GPX), and heme oxygenase-1 (HO-1) [37,42]. It also inhibits superoxide anion radical synthesis induced by homocysteine [43]. In patients with MAFLD, mitochondrial damage can occur with disease progression, leading to the development of NASH. This is a result of excessive β-oxidation occurring in the mitochondria due to the large amount of incoming fatty acids, resulting in excessive production of reactive oxygen species. Exposure of lipids to excessive oxidation results in the production of their toxic metabolites [15,44,45].

In addition, statistically significant decreases in ALT and AST levels were observed only by Panahi et al. [33] and Rahmani et al. [28], whose subjects were for 8 weeks supplemented with 3 × 500 mg/day (100 mg curcuminoids per capsule) and 500 mg/day of an amorphous dispersion preparation containing 70 mg curcuminoids, respectively. Chashmniam et al. [31], using phospholipid curcumin 250 mg/day (equivalent to 50 mg pure curcumin) for 8 weeks, also noted a decrease in ALT and AST levels, but did not report statistical significance. In other studies [29,30,32,34,35,36], non-statistically significant decreases in ALT levels were observed, regardless of the supplement used and the duration of supplementation (Kelardeh et al. [29]: 80 mg/day curcumin as nanomicelle, 12 weeks; Ghaffari et al. [30]: 3 g/day turmeric (6 × 500 mg/day), 12 weeks; Mirhafez et al. [32]: phospholipid curcumin 250 mg/day (equivalent to 50 mg pure curcumin), 8 weeks; Hariri et al. [34]: phospholipid curcumin 250 mg/day (equivalent to 50 mg pure curcumin), 8 weeks; Kelardeh et al. [35]: 80 mg/day curcumin as nanomicelle, 12 weeks; Saberi-Karimian et al. [36]: 500 mg curcuminoids plus 5 mg piperine/day, 8 weeks). Use of 3 g/day turmeric (6 × 500 mg/day) for 12 weeks in a study by Ghaffari et al. [30] led to a non-statistically significant increase in AST levels. In other studies (Kelardeh et al. [29]: 80 mg/day curcumin as nanomicelle, 12 weeks; Mirhafez et al. [32]: phospholipid curcumin 250 mg/day (equivalent to 50 mg pure curcumin), 8 weeks; Hariri et al. [34]: phospholipid curcumin 250 mg/day (equivalent to 50 mg pure curcumin), 8 weeks; Kelardeh et al. [35]: 80 mg/day curcumin as nanomicelle, 12 weeks; Saberi-Karimian et al. [36]: 500 mg curcuminoids plus 5 mg piperine/day, 8 weeks), there was a non-statistically insignificant decrease in AST levels.

Another important factor in the course of MAFLD is glucose. In studies comprising 8-week supplementation with 500 mg/day of an amorphous dispersion preparation comprising 70 mg curcuminoids (Rahmani et al.) [28]; phospholipid curcumin 250 mg/day (equivalent to 50 mg pure curcumin (Chashmniam et al. and Mirhafez et al.) [31,32], and 500 mg curcuminoids plus 5 mg piperine/day (Saberi-Karimian) [36] the results showed a decrease in the level of fasting glucose, but in three of these studies [28,32,36] the results were not statistically significant (Chashmniam et al. did not report statistical significance). Additionally, Rahmani et al. [28] reported a statistically significant decrease in HbA1c. Fasting glucose and HbA1c were not controlled. Lower levels of fasting glucose and HbA1c may have been the result of an improvement in the function of β-cells and an increase in insulin sensitivity. A study conducted among pre-diabetes patients using curcumin supplementation (3 × 250 mg of curcuminoids twice a day) for 9 months resulted in an improvement in the function of β-cells and a reduction in HOMA-IR, fasting glucose, and HbA1c levels [46]. A decrease in insulin resistance was also noted in a 4-week study among obese children taking 500 mg of curcumin daily [47].

Free fatty acids and free cholesterol also play an important role in the pathomechanism of MAFLD [15]. Chashmniam et al. [31] noted an increase in TG levels after the use of phospholipid curcumin at 250 mg/day (equivalent to 50 mg pure curcumin) (statistical significance not given). Rahmani et al. [28], Mirhafez et al. [32], and Saberi-Karimian et al. [36], using respectively for 8 weeks 500 mg/day of an amorphous dispersion preparation comprising 70 mg curcuminoids; phospholipid curcumin 250 mg/day (equivalent to 50 mg pure curcumin), and 500 mg curcuminoids plus 5 mg piperine/day, observed non-statistically significant decreases in TG levels. Only Panahi et al. [33], after the use of 3 × 500 mg/day (100 mg curcuminoids per capsule) in patients for 8 weeks, noted a statistically significant decrease in TG levels. The effectiveness of curcumin in reducing TG levels has also been observed in people with metabolic syndrome [48], obesity [49], and type-2 diabetes [50].

In the studies of Chashmniam et al. (phospholipid curcumin 250 mg/day (equivalent to 50 mg pure curcumin)) [31] and Saberi-Karimian et al. (500 mg curcuminoids plus 5 mg piperine/day) [36] after 8 weeks of supplementation, they reported increases in LDL-C levels (Chashmniam et al. do not provide information on statistical significance. Saberi-Karimian et al.: non-statistically significant change compared to the change in the control group). Mirhafez et al. (phospholipid curcumin 250 mg/day (equivalent to 50 mg pure curcumin)) [32] observed a statistically insignificant decrease in LDL-C levels after 8 weeks of supplementation, while Panahi et al. (3 × 500 mg/day (100 mg curcuminoids per capsule)) [33] and Rahmani et al. (500 mg/day of an amorphous dispersion preparation comprising 70 mg curcuminoids) [28] also recorded a statistically significant decrease after 8 weeks of supplementation.

Chashmniam et al. (phospholipid curcumin 250 mg/day (equivalent to 50 mg pure curcumin)) [31] and Panahi et al. (3 × 500 mg/day (100 mg curcuminoids per capsule)) [33] reported a decrease in HDL-C levels after 8 weeks of supplementation (Panahi et al. obtained a statistically significant result; Chashmniam et al. did not report statistical significance). Rahmani et al. (500 mg/day of an amorphous dispersion preparation comprising 70 mg curcuminoids) [28] also observed a statistically significant increase in the level of HDL-C after 8 weeks of supplementation. Mirhafez et al. (phospholipid curcumin 250 mg/day (equivalent to 50 mg pure curcumin)) [32] and Saberi-Karimian et al. (500 mg curcuminoids plus 5 mg piperine/day) [36] noticed statistically insignificant increases in HDL-C levels after 8 weeks of supplementation.

Rahmani et al. (500 mg/day of an amorphous dispersion preparation comprising 70 mg curcuminoids) [28], Chashmniam et al. [31] and Mirhafez et al. [32] (both phospholipid curcumin 250 mg/day (equivalent to 50 mg pure curcumin)), and Saberi-Karimian et al. (500 mg curcuminoids plug 5 mg piperine/day) [36] reported decreases in TC levels after 8 weeks of supplementation, but only Rahmani et al. [28] noted a statistically significant result. Panahi et al. (3 × 500 mg/day (100 mg curcuminoids per capsule)) [33] reported a statistically significant decrease in non-HDL-C levels. As shown by the studies carried out so far, curcumin exerts an effect on cholesterol metabolism. By increasing the expression of LDL receptors, it leads to an increase in the removal of LDL from the plasma and increased excretion of cholesterol in the bile. Curcumin is also responsible for the inhibition of Niemann-Pick C1-Like 1 (NPC1L1) protein. Moreover, curcumin also influences lipogenesis by inhibiting involved enzymes and receptors, including sterol regulators binding protein-1 element, apolipoprotein B-100, fatty acid synthase, acetyl CoA carboxylase, acyl coenzyme A, cholesterol acyltransferase, 3-hydroxy-3-methylglutaryl-coenzyme A reductase, peroxisome proliferator-activated receptor-α, cluster of differentiation 36, and adenosine monophosphate-activated protein kinase [28].

Uric acid is considered to be one of the factors involved in the MAFLD pathomechanism, as it exhibits pro-inflammatory and pro-oxidative effects by increasing the production of monocyte chemotactic protein-1 and activation of mitogen-activated protein kinase and nuclear factor κB pathways [51]. Panahi et al. [33] after using 3 × 500 mg/day (100 mg curcuminoids per capsule) in patients for 8 weeks, observed a statistically significant decrease in uric acid levels.

Mirhafez et al. (phospholipid curcumin 250 mg/day (equivalent to 50 mg pure curcumin)) observed a statistically significant decrease in leptin levels after 8 weeks of supplementation; a statistically significant increase in the level of adiponectin; and a statistically significant decrease in leptin:adiponectin ratio. This is important information in the context of future research, as increased levels of inflammatory cytokines, in addition to the factors mentioned above, may also be associated with leptin resistance, high leptin levels, and inhibition of adiponectin expression [32].

Considering that the curcumin content in turmeric is only a few percent and it also has a very low bioavailability after oral ingestion, in a study using a rat model, it was estimated at 1%. For this reason, supplements standardized for curcumin content are used for therapeutic purposes because the chances of obtaining a positive effect after using turmeric as a curcumin source are poor. Piperine from black pepper fruits can increase the bioavailability of curcumin by up to 2000% [52,53].

Our study has several strengths. First of all, only human studies were included. Studies with the exclusive use of curcumin supplementation were analyzed, without any other additional recommendations for patients, such as changing diet or lifestyle.

Because of differences in the study protocols and in the characteristics of supplemented patient cohorts in terms of age, BMI, and disease progression, more studies on the efficacy of curcumin supplementation in MAFLD patients of different ages and at different stages of disease progression should be conducted. In addition, further studies could compare effects of different dosages of curcumin supplementation and effects of various supplementation periods.

## 5. Conclusions

Curcumin has some therapeutic potential in MAFLD. However, the studies conducted so far do not allow us to unequivocally determine the positive effects of its action. It is also impossible to establish an effective dosing regimen. For these reasons it is necessary to conduct further studies using larger groups of patients and different doses of the supplement.

## Figures and Tables

**Table 1:** PICOS criteria for inclusion and exclusion of studies.

Parameter	Defined Criteria for the Current Study
P (population)	Adult patients with MAFLD
I (intervention	Curcumin supplementation
C (comparison)	No special comparison criteria
O (outcomes)	Changes in biochemical parameters
S (study design)	Any type apart from case reports and reviews

**Table 2:** Characteristics of studied groups.

	n	Age (Year)	BMI (kg/m^2^)
Rahmani, 2016 [21]	40 (19 men, 21 women) *	46.37 ± 11.57	30.84 ± 4.45
Kelardeh, 2017 [22]	12 men	37.41 ± 5.17	29.88 ± 4.49
Ghaffari, 2018 [23]	21 (11 men, 10 women)	42.57 ± 6.93	31.81 ± 4.58
Chashmniam, 2019 [24]	25 (13 men, 12 women)	46.56 ± 2.25	30.03 ± 0.7
Mirhafez, 2019 [25]	32 (18 men, 14 women)	44.8 ± 11.14	30.06 ± 5.76
Panahi, 2019 [26]	36 (19 men, 17 women)	49.4 ± 8.7	27.6 ± 2.5
Hariri, 2020 [27]	23 (14 men, 9 women)	40.95 ± 12.24	30.59 ± 5.91
Kelardeh, 2020 [28]	11 women	66.72 ± 3.03	27.60 ± 1.26
Saberi-Karimian, 2020 [29]	26	18-70	30.02 ± 5.45

* 3 drop-outs before final analysis.

**Table 3:** Characteristics of included studies.

	Study Design	Dose	Duration	n (Study Group)	n (Control Group)	Tested Parameters
Rahmani, 2016 [28]	RCT	500 mg/day of an amorphous dispersion preparation comprising 70 mg curcuminoids	8 weeks	37	40	ALT, AST, TG, TC, LDL-C, HDL-C, FBG, HbA1c
Kelardeh, 2017 [29]	RCT	80 mg/day curcumin as nanomicelle	12 weeks	12	12	ALT, AST, ALP
Ghaffari, 2018 [30]	DB, RCT	3 g/day turmeric (6 × 500 mg/day)	12 weeks	21	21	ALT, AST, MDA, TAC, IL-6, hs-CRP, TNF-α
Chashmniam, 2019 [31]	DB, RCT	Phospholipid curcumin 250 mg/day (equivalent to 50 mg pure curcumin)	8 weeks	25	20	ALT, AST, ALP, FBS, TC, TG, LDL-C, HDL-C, T Bili, D Bili, Creat, Urea
Mirhafez, 2019 [32]	DB, RCT	Phospholipid curcumin 250 mg/day (equivalent to 50 mg pure curcumin)	8 weeks	32	29	ALT, AST, TG, TC, LDL-C, HDL-C, FBS, Leptin, Adiponectin, Leptin:Adiponectin
Panahi, 2019 [33]	CT	3 × 500 mg/day (100 mg curcuminoids per capsule)	8 weeks	36	-	ALT, AST, TG, LDL-C, HDL-C, Non-HDL-C, Uric acid
Hariri, 2020 [34]	DB, RCT	Phospholipid curcumin 250 mg/day (equivalent to 50 mg pure curcumin)	8 weeks	23	22	ALT, AST
Kelardeh, 2020 [35]	RCT	80 mg/day curcumin as nanomicelle	12 weeks	11	11	ALT, AST
Saberi-Karimian, 2020 [36]	RCT	500 mg curcuminoids + 5 mg piperine/day	8 weeks	23	26	ALT, AST, TG, TC, LDL-C, HDL-C, FBG, IL-2, IL-4, IL-6, IL-8, IL-10, VEGF, IFNγ, TNF-α, IL-1α, IL-1β, MCP-1, EGF

ALP, alkaline phosphatase; ALT, alanine aminotransferase; AST, aspartate aminotransferase; Creat, creatinine; CT, clinical trial; DB, double blind; D Bili, direct bilirubin; EGF, epidermal growth factor; FBG, fasting blood glucose; HbA1c, glycated hemoglobin; HDL-C, high-density lipoprotein cholesterol; hs-CRP, high-sensitivity C-reactive protein; IFNγ, interferon gamma; IL-1α, interleukin-1 alpha; IL-1β, interleukin-1 beta; IL-2, interleukin-2; IL-4, interleukin-4; IL-6, interleukin-6; IL-8, interleukin-8; IL-10, interleukin-10; LDL-C, low-density lipoprotein cholesterol; MCP-1, monocyte chemoattractant protein-1; MDA, malondialdehyde; non-HDL-C, non-high-density lipoprotein cholesterol RCT, randomized controlled trial; TAC, total antioxidant capacity; T Bili, total bilirubin TC, total cholesterol; TG, total triglycerides; TNF-α, tumor necrosis factor alpha; VEGF, vascular endothelial growth factor.
